# Capturing Real-World Habitual Sleep Patterns With a Novel User-Centric Algorithm to Preprocess Fitbit Data in the All of Us Research Program: Retrospective Observational Longitudinal Study

**DOI:** 10.2196/71718

**Published:** 2025-07-28

**Authors:** Hiral Master, Jeffrey Annis, Jack H Ching, Karla Gleichauf, Lide Han, Peyton Coleman, Kelsie M Full, Neil Zheng, Douglas Ruderfer, John Hernandez, Logan D Schneider, Evan L Brittain

**Affiliations:** 1 Vanderbilt University Medical Center Nashville, TN United States; 2 Google Mountain View, CA United States; 3 Brigham and Women's Hospital Boston, MA United States

**Keywords:** sleep, Fitbit, algorithms, All of Us Research Program, metrics, R package

## Abstract

**Background:**

Commercial wearables such as Fitbit quantify sleep metrics using fixed calendar times as default measurement periods, which may not adequately account for individual variations in sleep patterns. To address this limitation, experts in sleep medicine and wearable technology developed a user-centric algorithm designed to more accurately reflect actual sleep behaviors and improve the validity of wearable-derived sleep metrics.

**Objective:**

This study aims to describe the development of a new user-centric algorithm, compare its performance with the default calendar-relative algorithm, and provide a practical guide for analyzing All of Us Fitbit sleep data on a cloud-based platform.

**Methods:**

The default and user-centric algorithms were implemented to preprocess and compute sleep metrics related to schedule, duration, and disturbances using high-resolution Fitbit sleep data from 8563 participants (median age 58.1 years, 6002/8341, 71.96%, female) in the All of Us Research Program (version 7 Controlled Tier). Variations in typical sleep patterns were calculated by examining the differences in the mean number of primary sleep logs classified by each algorithm. Linear mixed-effects models were used to compare differences in sleep metrics across quartiles of variation in typical sleep patterns.

**Results:**

Out of 8,452,630 total sleep logs collected over a median of 4.2 years of Fitbit monitoring, 401,777 (4.75%) nonprimary sleep logs identified by the default algorithm were reclassified as primary sleep by the user-centric algorithm. Variation in typical sleep patterns ranged from –0.08 to 1. Among participants with the greatest variation in typical sleep patterns, the user-centric algorithm identified significantly more total sleep time (by 17.6 minutes; *P*<.001), more wake after sleep onset (by 13.9 minutes; *P*<.001), and lower sleep efficiency (by 2.0%; *P*<.001), on average. Differences in sleep stage metrics between the 2 algorithms were modest.

**Conclusions:**

The user-centric algorithm captures the natural variability in sleep schedules, providing an alternative approach to preprocess and evaluate sleep metrics related to schedule, duration, and disturbances. A publicly available R package facilitates the implementation of this algorithm for clinical and translational research.

## Introduction

Consumer wearables such as Fitbits are increasingly used in research studies to investigate the impact of biomarkers and lifestyle behaviors on health outcomes, supporting applications ranging from screening to health management. A leading example is the National Institutes of Health–funded All of Us Research Program, which collects longitudinal Fitbit data with privacy-preserving linkage to electronic health records (EHRs) and other data types [1,2]. Studies based on this data set have documented associations between Fitbit-derived activity and sleep metrics and the onset of chronic diseases [3,4]. The growing interest in wearables research has also led to the development of recent industry standards documenting best practices for validating and using digital health end points related to physical activity [5,6], heart rate [7], and sleep patterns [8]. In parallel, researchers have also developed preprocessing methods for activity to account for biases and limitations inherent to wearable-measured data in real-world settings [9,10]. Recent-generation Fitbit devices, which employ heart rate variability and limb movement, have demonstrated promising performance in measuring standard sleep metrics such as wake after sleep onset (WASO) and total sleep time (TST), and in classifying sleep versus wake episodes more accurately than clinical actigraphy, which relies solely on limb movement [11,12]. Additional research is still needed, however, to develop a diverse toolkit for preprocessing and validating wearable-derived sleep metrics [13], especially for populations underrepresented in data sets used for algorithm development, such as older adults and individuals with multiple chronic health conditions [14]. This limitation has prompted professional clinical associations to advise caution in the use of such devices in clinical practice [15].

Sleep metrics accessible to researchers via the Fitbit Web application programming interface (API) currently rely on a default sleep algorithm, isMainSleep, which was primarily designed for general consumer use. By default, this algorithm uses the calendar date to calculate summary sleep metrics based on primary sleep logs—defined as the set of logs corresponding to a user’s main sleep period—classified as those with the longest duration ending on each calendar day. While this approach generally performs well, the isMainSleep algorithm does not fully capture primary sleep periods that include interruptions involving at least an hour of wakefulness. Such interruptions result in the creation of multiple sleep logs, with only the longest log reported as the primary sleep period [16].

Burgess et al [13] have elucidated similar limitations and developed an automated sleep data cleaning method that requires Fitbit sleep data from a third-party platform (Fitabase) and self-reported sleep diaries. In this study, however, in collaboration with clinical and technical experts in sleep medicine from industry and academia, we propose a fully data-driven algorithm that does not require self-reported sleep logs or manual scoring for preprocessing Fitbit sleep data, making it more scalable for sleep studies across large populations. Specifically, we propose a user-centric algorithm based on an individual’s typical sleep period (TSP), which accounts for variability in habitual sleep patterns and has the potential to reduce noise in sleep data and more accurately capture sleep-wake metrics (as shown in Multimedia Appendix 1). This paper details the development of the TSP algorithm, compares it with the default calendar-relative isMainSleep algorithm, and offers a practical guide for analyzing All of Us Fitbit sleep data on a cloud platform. We also examine the characteristics of participants whose sleep behaviors deviate from typical sleep patterns and make the analytic pipelines publicly available—through an open-source R package—to support the implementation of Fitbit sleep data processing for clinical and research use.

## Methods

### Study Design and Population

A retrospective observational longitudinal study using secondary data analysis was conducted to implement the algorithms for preprocessing Fitbit sleep data. We used deidentified data from All of Us participants, made available to researchers in the version 7 controlled tier (C2022Q4R13) on the All of Us Researcher Workbench—a secure, cloud-based platform [17]. A detailed description of this program has been published elsewhere [2]. Data for this study were derived from a convenience sample of participants who owned a Fitbit device and were able to contribute their Fitbit data since creating a Fitbit account under the “Bring Your Own Device” Program. Specifically, we included 8563 participants who provided EHR data and had any Fitbit sleep data (described below) from 2009 to 2022.

### Ethical Considerations

The protocol for the human participant research conducted was reviewed by the Institutional Review Board of the All of Us Research Program (protocol 2021-02-TN-001). Data security and participant confidentiality were safeguarded through multiple measures: secure data storage systems were implemented, access to identifying information was restricted, and confidentiality protocols were mandated through contractual agreements. Access to the Researcher Workbench was limited to authorized personnel who had completed mandatory training and whose institutions were covered by active data use agreements. Compensation of US $25 was provided via a cash payment, gift card, or electronic voucher when biological specimens (blood, saliva, or urine) were collected at designated partner facilities. To protect participant privacy, in accordance with the All of Us Research Program’s Data and Statistics Dissemination Policy, values corresponding to a small number of participants (n<20), or any values from which such small sample sizes could be deduced using mathematical formulas, were not reported.

In this study, only the authorized authors who completed the All of Us Responsible Conduct of Research training accessed the deidentified data via the Researcher Workbench, a secure cloud-based platform. As the authors were not directly involved with the participants, institutional review board review was exempted.

### All of Us Fitbit Sleep Data

Deidentified daily summaries of Fitbit sleep data, as well as higher-resolution sequence-level sleep data obtained via the Fitbit Web API, were made available on the Researcher Workbench through a multistep data ingestion and curation process detailed elsewhere [1]. While Fitbit provides additional data on short wake periods (representing physiological awakenings of 3 minutes or less), the TSP algorithm was implemented using only the sequence-level data currently available on the All of Us Researcher Workbench. This data set captures sleep periods of at least 1 hour and intervening wake periods during sleep greater than 3 minutes. The sequence-level sleep data also include a detailed breakdown of sleep periods into either accelerometry-derived classic (nonstaged) sleep patterns (ie, awake, restless, asleep) or sleep stages (ie, wake, light, deep, and rapid eye movement [REM]); the latter are available for sleep periods of 3 hours or longer and are derived using heart rate data. These Fitbit data were retrospective and longitudinal in nature and included all available data since the time participants created their Fitbit accounts.

### Resolutions of Fitbit Sleep Data

For ease of exposition, we first define the different resolutions at which Fitbit sleep data can be analyzed. At the broadest level, sleep can be conceptualized as an entire sleep period. A single sleep period may consist of 1 or more sleep logs, including any interruptions that occur—defined as intervening wakefulness of ≥1 hour between sleep logs.

Each sleep log consists of a consecutive sequence of sleep segments separated by less than 1 hour and may be classified as either primary or nonprimary sleep. Each sleep segment within a log is associated with a sleep level and includes a start time stamp and duration. The sleep level indicates whether the segment represents a period of sleep—categorized as “asleep” in Fitbit’s classic sleep pattern algorithm, or as “light,” “deep,” or “REM” in the sleep staging algorithm—or a period of wakefulness—categorized as “awake” or “restless” in the classic algorithm, or “wake” in the sleep staging algorithm.

### Primary Sleep Period

In this paper, we introduce the term primary sleep period to refer to the time during which a typical person obtains the majority of their sleep. Accurate identification of the primary sleep period is particularly important for assessing related sleep-wake metrics, as shown in Multimedia Appendix 1. Most canonical “regular” sleepers with a daytime schedule will have primary sleep periods between 11:00 PM and 7:00 AM. However, some individuals have less conventional sleep patterns. For example, a night shift worker may aim to sleep immediately after work (eg, 8:00 AM to 1:00 PM) and then take a nap between 5:00 PM and 7:00 PM. Conceptually, we would label the hours between 8:00 AM and 1:00 PM as their primary sleep period for that day, and any time outside this window as their nonprimary sleep period. In this instance, the time between 5:00 PM and 7:00 PM would be counted as TST during the nonprimary sleep period.

A second example involves participants with significant disruptions in their sleep—for instance, someone who falls asleep from 10:00 PM to 11:30 PM, is awake in bed until 1:30 AM, and then sleeps again until 6:30 AM. In such cases, we consider it reasonable to define their primary sleep period as spanning from 10:00 PM to 6:30 AM, despite the prolonged 2-hour period of wakefulness. The underlying assumption for stitching these 2 disjointed sleep logs into a single (10:00 PM-6:30 AM) sleep period under the user-centric algorithm is that if a sleep log is at least 1 hour in duration and either overlaps with the TSP or ends within 1 hour of sleep occurring during the TSP, it likely reflects an intent to sleep. In such cases, the sleep log should be included in the primary sleep period, even if a significant intervening wakefulness period (eg, due to insomnia) is present. Additionally, it may be appropriate to include such sleep in the primary sleep period, given its relevance to sleep health. To clarify, inadvertent dozing rarely lasts longer than 1 hour—especially during the zone of maximum circadian drive for wakefulness, which typically occurs just before habitual bedtime. Therefore, it is reasonable to treat such sleep segments as part of the primary physiological or behavioral sleep period, to capture unhealthy sleep habits and provide a more holistic view of sleep architecture.

### Primary Sleep Log Classification Under isMainSleep and TSP Algorithms

**Figure 1:**
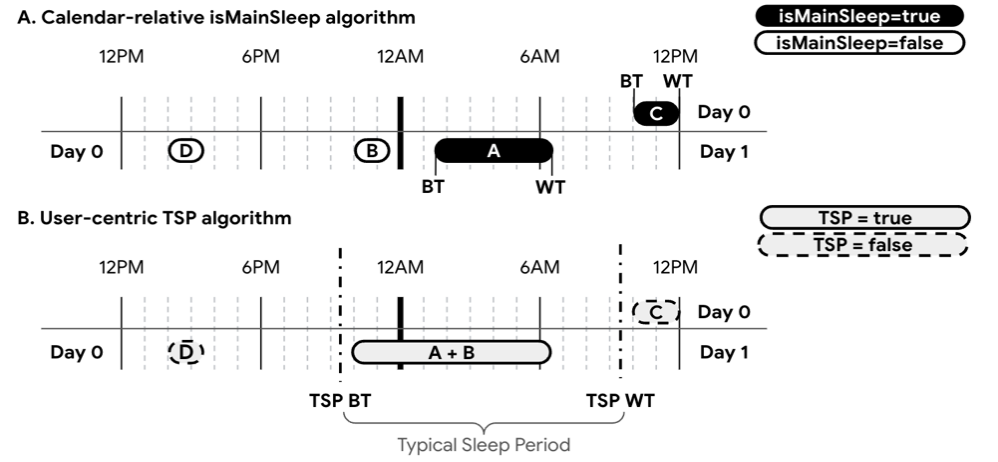
Conceptual overview of how the (A) calendar-relative “isMainSleep” and (B) user-centric “TSP” algorithms handle high-resolution sleep data. TSP BT and TSP WT are derived from the median bedtime and wake time across all relevant sleep logs around a user’s median midsleep point (MSP) (not shown). BT: bedtime; TSP: typical sleep period; WT: wake time.

An individual’s sleep may be fragmented and distributed across time. In the conceptual example illustrated in Figure 1, there are 4 sleep logs spanning 2 calendar days: C (day 0; 10:00 AM-noon), D (day 0; 2:00-3:30 PM), B (day 0; 10:00-11:30 PM), and A (day 1; 1:30-6:30 AM). To accurately compute sleep metrics, it is imperative to identify and include all primary sleep logs that fall within the primary sleep period, while distinguishing them from nonprimary sleep logs.

As shown in Figure 1A, isMainSleep classifies sleep logs C and A as the primary sleep logs on days 0 and 1, respectively, since they are the longest-duration sleep logs on each respective midnight-to-midnight calendar day. By contrast, the TSP algorithm (described in detail below) adopts a user-centric approach that is agnostic to time-of-day (ie, it does not rely on a fixed calendar-based time window). TSP classifies both sleep logs A and B as primary sleep on day 1 (Figure 1B). It also reclassifies sleep log C as nonprimary sleep on day 0, as it falls outside the TSP. Sleep log D, which is neither the longest-duration log on day 1 nor overlapping with the TSP, is classified as nonprimary sleep by both the isMainSleep and TSP algorithms.

### Real-World Data Example

**Figure 2:**
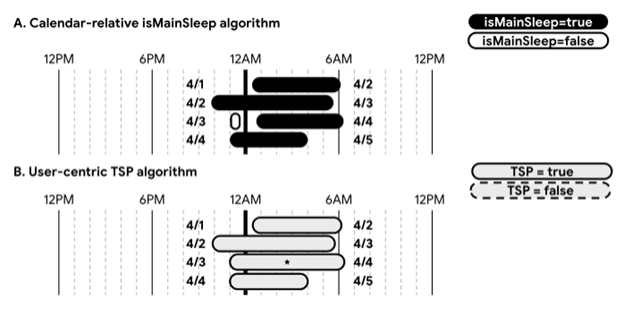
(A) Calendar-relative ‘isMainSleep’ versus (B) user-centric ‘TSP’ algorithm—an illustrative data example. TSP: typical sleep period. The data shown in this figure are sourced directly from the Fitbit Web application programming interface sleep records for one of the co-authors (LS), who has consented to share his data for illustration purposes. Nonprimary sleep logs are not displayed in the visualization, as none were recorded during this time frame.

Figure 2 provides an illustrative example of a limitation of the isMainSleep algorithm, using actual Fitbit-measured sleep data from one of the co-authors (LS), who has consented to share his data. In this example, the primary sleep period spanning April 3 to April 4 includes 2 sleep logs, with one starting on April 3 and the other ending on April 4. The isMainSleep algorithm classifies only the longer sleep log on April 4 as primary sleep and misclassifies the initial sleep log on April 3 as nonprimary. As a result, compared with the full primary sleep period, isMainSleep incorrectly reports a much later bedtime, shorter time attempting to sleep (TATS), TST, and WASO, as well as fewer number of awakenings (NWAK) and an artificially higher sleep efficiency percentage.

In contrast to isMainSleep, the TSP algorithm classifies both sleep logs—along with the intervening wake period between them—as part of the primary sleep period spanning April 3 to April 4 (asterisk in Figure 2). Notably, the primary sleep periods ending on April 2, April 3, and April 5 are correctly classified by the isMainSleep algorithm, as they correspond to the longest-duration sleep logs ending on each calendar day; thus, there is no difference in classification under TSP for those days.

### Technical Implementation of Both Algorithms

**Figure 3:**
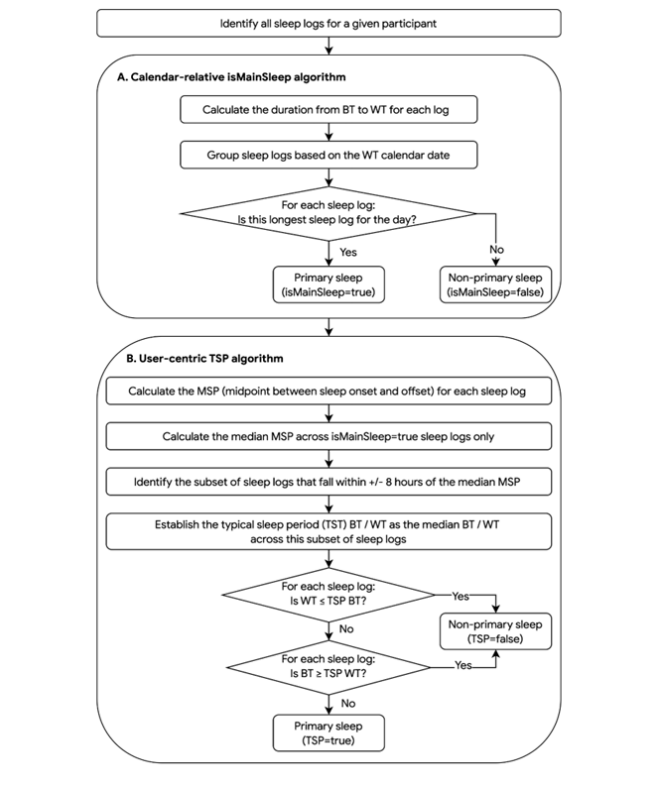
Decision-making process for sleep log classification using (A) calendar-relative and (B) user-centric algorithms. BT: bed time; MSP: midsleep point; TSP: total sleep period; WT: wake time.

The decision-making process for classifying sleep logs using both algorithms is illustrated in Figure 3A, B. The calendar-relative isMainSleep algorithm identifies primary sleep as the longest sleep log on each calendar day (Figure 3A). By contrast, the user-centric TSP algorithm is agnostic to calendar dates and aggregates sleep logs based on a user’s TSP through a multistep process. The algorithm first determines the typical time range during which the user records their longest sleep each day and then identifies as primary sleep any logs that overlap with this range (Figure 3B; detailed in Multimedia Appendix 2).

### Variation From Typical Sleep Patterns

To assess how much a participant’s sleep patterns deviate from the assumptions of the calendar-relative isMainSleep algorithm, we apply the TSP algorithm and compute the difference in the number of primary sleep logs classified by TSP versus isMainSleep per night for each participant (equation 1). Depending on how individual sleep logs are reclassified by TSP, the number of primary sleep logs assigned to a given night may increase, decrease, or remain unchanged.

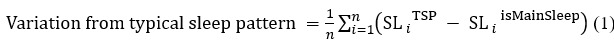



for the *i*th day in a monitoring period of *n* total days given the number of sleep logs SL.

For primary sleep periods that include 2 or more sleep logs, this measure increases when disjointed logs are “stitched” together by TSP to represent the full primary sleep period (eg, an increase from 1 to 2 logs on day 1 in Figure 1). Conversely, for primary sleep periods in which an isMainSleep-classified primary sleep log falls outside of the participant’s TSP, the measure decreases when these logs are “cleaned” by TSP and reclassified as naps (eg, a decrease from 1 to 0 logs on day 0 in Figure 1). When neither stitching nor cleaning is applied, there is no change in the number of primary sleep logs. Thus, the net change in the mean number of primary sleep logs per night reflects the nature of a participant’s sleep: a net increase under TSP suggests fragmented or interrupted primary sleep, a net decrease indicates sleep occurring outside of the TSP, and no change implies consistency with typical sleep patterns.

### Demographic and Clinical Characteristics

Demographic characteristics such as age, sex at birth, race/ethnicity, and education were self-reported by participants through the Basics survey at the time of enrollment. EHR data from multiple health care provider organizations—funded and participating in the All of Us Program—were harmonized using the Observational Medical Outcomes Partnership common data model [18,19]. These harmonized EHR data were used to derive BMI and identify incident insomnia cases, which were quantified using International Classification of Diseases billing codes mapped to phecodes, as described elsewhere [20,21]. To determine incident insomnia cases, any diagnosis occurring before or during the first 6 months of Fitbit sleep monitoring was excluded from consideration as an “incident case” in this study.

### Analysis

Descriptive statistics for participant characteristics were summarized using the median and IQR for continuous variables, and frequencies for categorical variables. Mann-Whitney *U* tests and chi-square tests were used to compare continuous and categorical variables, respectively, between participants included and excluded from the analytical data set. The isMainSleep and TSP algorithms were applied to sequence-level Fitbit data on the Researcher Workbench using the R programming language (R Foundation) [22]. The number of primary versus nonprimary sleep logs identified by the 2 algorithms was evaluated using a 2×2 contingency table.

A data-driven quartile approach was used to empirically examine variation from typical sleep patterns across clinical and demographic characteristics, as well as sleep onset/offset patterns. Sleep metrics (listed in Multimedia Appendix 1) were then quantified using both algorithms across these quartiles. To account for repeated measures within individuals and interindividual variability in algorithm impact, mixed-effects linear regression models were used to evaluate the main effects (ie, quartiles and algorithms) and their interaction. Specifically, these models were used to assess how sleep metrics—such as TATS, TST, WASO, NWAK, TST in the nonprimary sleep period, counts of nonprimary sleep episodes, total wakefulness duration, and sleep efficiency percentage—differ between the isMainSleep and TSP algorithms. Post hoc pairwise comparisons were conducted using the Wilcoxon signed rank test. To assess the robustness of findings, differences in sleep metrics across algorithms were also evaluated among participants with a higher proportion of “cleaned” versus “stitched” sleep logs.

Given the longitudinal nature of the Fitbit sleep data, we additionally examined whether our findings were sensitive to the amount of sleep data contributed by participants. Sensitivity analyses were conducted by excluding participants who had fewer than 4 hours of recorded sleep data on more than 30% of days. These thresholds were informed by prior mathematical models identifying the theoretical cutoff below which insufficient sleep would be considered unsustainable [23].

### Code Availability

The code used for this study is available to approved researchers on the All of Us Researcher Workbench platform upon request by contacting the study team. Additionally, the R package and the code used to generate the results shown in the illustrative example (Figure 2) are publicly available on GitHub [24].

## Results

### Overview

Of the 413,457 total participants in the version 7 Controlled Tier on the Researcher Workbench, 8563 (6002/8341, 71.96%, female and 7068/8008, 88.26%, White) provided both sleep and EHR data and were included in the analytical sample (Table 1). The median duration of Fitbit sleep monitoring was 4.2 years and included sleep data collected over weekends.

**Table 1:** Participant characteristics for those included and excluded from the analytical cohort.

Descriptive statistics			Included (n=8563)		Excluded^a^ (n=404,894)		*F* test (*df*) or chi-square (*df*); *P* value
Age (years), median (IQR)			58.1 (43.1-69.1)		57.1 (41.1-69.1)		15.6 (1, 413,455)^b^; <.001
**Sex at birth, n/N (%)**							382 (1)^c^; <.001
	Female	6002/8341 (71.96)		243,563/396,393 (61.44)			
	Male	2339/8341 (28.04)		152,830/396,393 (38.56)			
**Race, n/N (%)**							1655 (2)^c^; <.001
	Black	454/8008 (5.67)		78,050/328,988 (23.72)			
	Other	486/8008 (6.07)		28,848/328,988 (8.77)			
	White	7068/8008 (88.26)		222,090/328,988 (67.51)			
**Education, n/N (%)**							2779 (2)^c^; <.001
	College	5955/8332 (71.47)		176,390/391,595 (45.04)			
	No college	509/8332 (6.11)		112,937/391,595 (28.84)			
	Some college	1868/8332 (22.42)		102,268/391,595 (26.12)			
BMI (kg/m^2^), median (IQR)			28.4 (24.7-33.5)		28.3 (24.4-33.6)		6.35 (1, 331,488)^b^; .01
Fitbit monitoring duration (years), median (IQR)			4.2 (2.2-6.3)		4.0 (1.9-6.2)		11.6 (1, 14,906)^b^; <.001

^a^Participants were excluded because of not having electronic health records and Fitbit sleep data. The total number of participants for demographic characteristics such as sex at birth, race, ethnicity, and education are different from the primary analytical sample size because participants who have missing responses such as “none indicated” or “skip” have been removed.

^b^*F* test.

^c^Chi-square test.

### Impact of Algorithms on Sleep Logs and Participant Characteristics

Most sleep logs in categories A and D indicated “no change” using either algorithm (7,870,306/8,435,172, 93.30%; Table 2), suggesting that these logs were consistently classified as either primary or nonprimary sleep by both algorithms. However, 401,777 (4.76%) sleep logs in category B were classified as nonprimary sleep under isMainSleep but reclassified as primary sleep under TSP after “stitching.” A total of 180,547 (2.14%) sleep logs in category C, classified as primary sleep under isMainSleep, actually fall outside the TSP and were reclassified as nonprimary sleep via “cleaning.” The mean change in primary sleep logs per night after applying TSP—which measures variation from typical sleep patterns—ranged from –0.076 to 1 (Multimedia Appendix 3). Negative values represent a relatively larger net “cleaning” effect, where TSP reduced the mean number of primary sleep logs per night among participants whose nonprimary sleep was incorrectly labeled as primary sleep under isMainSleep. Values between 0 and 0.05 indicate a relatively modest net impact of TSP, reflecting consistent classification of sleep logs across both algorithms. Values greater than 0.05 represent a relatively larger net “stitching” effect, where TSP increased the mean number of primary sleep logs per night among participants with interrupted sleep that was incompletely captured under isMainSleep.

**Table 2:** 2×2 matrix table of primary sleep logsa as classified by calendar-relative and user-centric algorithms.

Number of sleep logs^a^			Calendar-relative (isMainSleep) algorithm					
			Primary sleep “isMainSleep=true”		Nonprimary sleep “isMainSleep=false”			

**User-centric (TSP^b^) algorithm, n (%)**								
	Primary Sleep “TSP=true”	A (no change): 7,132,635 (84.56)		B (stitching): 401,777 (4.76)				
	Non-Primary Sleep “TSP=false”	C (cleaning): 180,547 (2.14)		D (no change): 737,671 (8.75)				

^a^The sum of logs in each category exceeds the total sleep logs (N=8,435,172) due to 17,458 sleep logs reporting more than 1 value for isMainSleep. This discrepancy arises from the simplified method used to reconstruct sleep logs, as the log ID from the Fitbit Web application programming interface was not available in the data set at the time of analysis on the Researcher Workbench.

^b^TSP: typical sleep period.

Table 3 presents participant characteristics stratified by variation in typical sleep patterns. Quartile 1 (Q1) represents participants with the lowest variation, while Q4 includes those with the highest variation—characterized by a greater proportion of “cleaned” and “stitched” logs. Compared with participants in Q1, those in Q4 are older (age: mean 61.1 years, SD 14.7 years vs mean 54.5 years, SD 15.5 years) and have a higher incidence of insomnia (19% vs 10%).

**Table 3:** Characteristics of participants across quartiles of variation from typical sleep patterns.

Characteristics				Quartile 1 (n=2140)		Quartile 2 (n=2141)		Quartile 3 (n=2141)		Quartile 4 (n=2141)		
Change in the number of primary sleep logs (TSP^a^—isMainSleep) per night, range for each IQR				–0.08 to 0.01		0.01 to 0.02		0.02 to 0.06		0.06 to 1		
**Descriptive statistics**												
	Age (years), median (IQR)			56.2 (41.2-67.2)		55.2 (41.2-67.2)		58.2 (44.2-69.2)		64.2 (50.2-73.2)		
	**Sex at birth, n/N (%)**											
		Female	1491/2078 (71.75)		1530/2098 (72.93)		1540/2088 (73.75)		1441/2077 (69.38)			
		Male	587/2078 (28.25)		568/2098 (27.07)		548/2088 (26.25)		636/2077 (30.62)			
	**Race, n/N (%)**											
		Black	71/1985 (3.58)		85/2021 (4.21)		132/2006 (6.58)		166/1996 (8.32)			
		Other	114/1985 (5.74)		148/2021 (7.32)		120/2006 (5.98)		104/1996 (5.21)			
		White	1800/1985 (90.68)		1788/2021 (88.47)		1754/2006 (87.44)		1726/1996 (86.47)			
	**Education, n/N (%)**											
		College	1566/2077 (75.40)		1581/2095 (75.47)		1448/2085 (69.45)		1360/2075 (65.54)			
		No college	107/2077 (5.15)		105/2095 (5.01)		136/2085 (6.52)		161/2075 (7.76)			
		Some college	404/2077 (19.45)		409/2095 (19.52)		501/2085 (24.03)		554/2075 (26.70)			
	BMI (kg/m^2^), median (IQR)			27.2 (23.7-31.5)		27.7 (24.3-32.5)		29.0 (25.2-33.9)		30.1 (26.0-35.7)		
	Fitbit monitoring duration (years), median (IQR)			3.4 (1.6-5.5)		4.7 (2.6-6.49)		4.7 (2.5-6.5)		4.0 (2.0-6.3)		
	Incident insomnia diagnosis, n/N (%)			221/2107 (10.49)		220/2105 (10.45)		305/2104 (14.50)		389/2083 (18.67)		

^a^TSP: typical sleep period.

### Sleep Metrics

**Figure 4:**
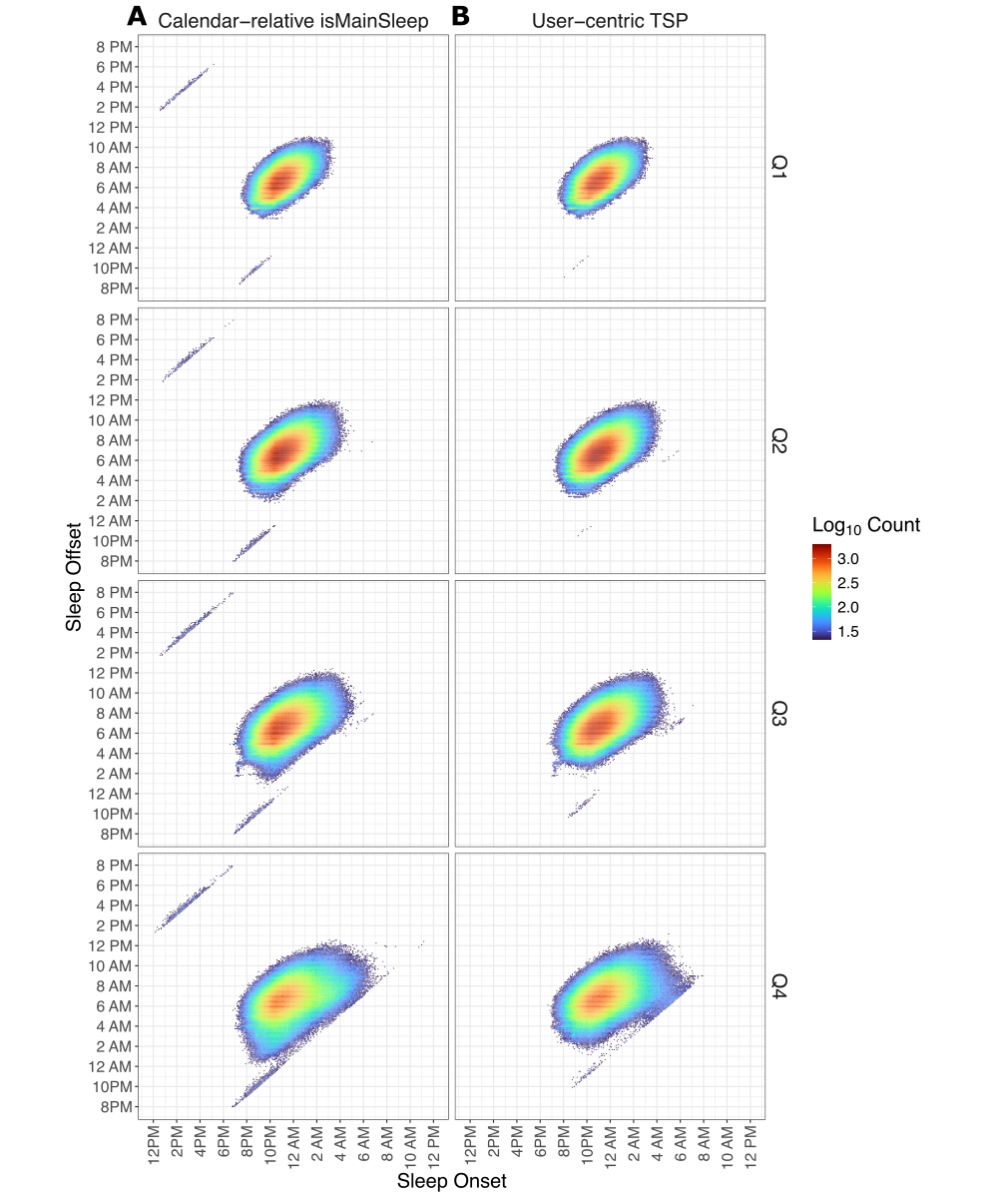
Sleep onset offset by quartiles of variation from typical sleep patterns using (A) calendar-relative and (B) user-centric algorithms.

Visual inspection of the sleep onset and offset heatmaps (Figure 4A, B) reveals that the TSP algorithm effectively reclassifies all isMainSleep-identified primary sleep logs that fall outside the midnight to noon window across all quartiles. This is evidenced by a marked reduction in short sleep logs occurring around midday—likely nonprimary sleep episodes misclassified as primary by isMainSleep. Additionally, there are notable differences in median sleep onset and offset times between the 2 algorithms, with greater variability observed among participants in higher quartiles of deviation from typical sleep patterns. Among Q4 participants, sleep onset and offset times were shifted by 6 and 37 minutes, respectively, compared with 5 and 15 minutes among Q1 participants.

**Figure 5:**
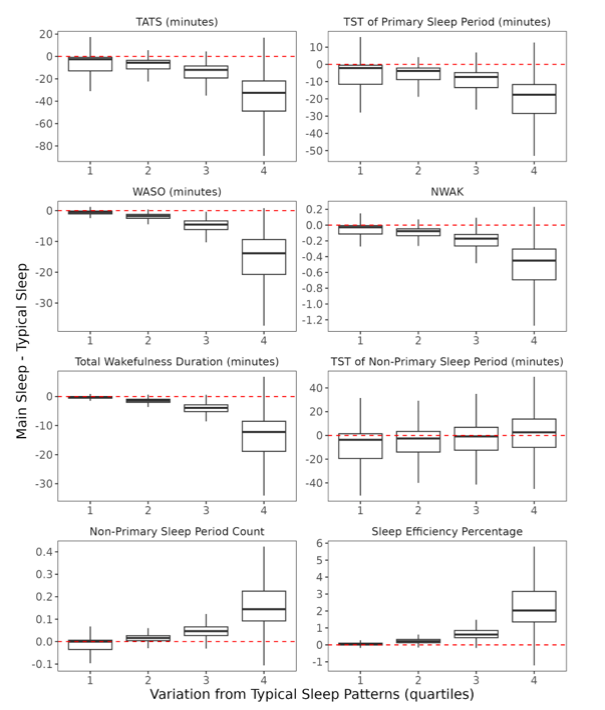
Boxplots of paired differences between user-centric (TSP) and calendar-relative (isMainSleep) algorithms for each of the key hypothesized sleep metrics across the quartiles of variation from typical sleep patterns. Box bounds, midline, and whiskers represent the IQR, median, and 1.5 × IQR, respectively. Values above the dotted red line indicate a higher value from the isMainSleep algorithm relative to the TSP algorithm, whereas values below the red line indicate a lower value. NWAK: number of awakenings; TATS: time attempting to sleep; TST: total sleep time; WASO: wake after sleep onset.

Figure 5 and Multimedia Appendix 4 show significant interactions between the algorithm applied and all key sleep metrics across quartiles (*P*<.001). Among Q4 participants, the median (IQR) TATS was 404.72 (IQR 350.4-447.91) minutes under isMainSleep, compared with 442.03 (IQR 399.17-482.93) minutes under TSP (Multimedia Appendix 4). This indicates that isMainSleep underestimated median TATS by 32.5 minutes (IQR 21.9-48.8; *P*<.001) relative to TSP. Similar underestimations were observed for other sleep metrics when computed using isMainSleep: TST by 17.6 minutes (IQR 11.7-28.4; *P*<.001), WASO by 13.9 minutes (IQR 9.4-20.7; *P*<.001), NWAK by 0.5 awakenings (IQR 0.3-0.7; *P*<.001), and total wakefulness duration by 12.2 minutes (IQR 8.5-18.9; *P*<.001). By contrast, among Q4 participants, isMainSleep-derived median nonprimary sleep counts (0.31, IQR 0.22-0.46) and sleep efficiency percentage (91.27, IQR 89.66-92.67) were significantly higher than those derived using TSP: median 0.15 (IQR 0.07-0.27) and median 88.96 (IQR 86.71-90.51), respectively. The differences—median 0.14 (IQR 0.09-0.22) for nonprimary sleep counts and median 2.0% (IQR 1.35%-3.2%) for sleep efficiency—were statistically significant (*P*<.001). Similar trends were observed across Q1-Q3, though the magnitude of differences was attenuated relative to Q4 (Multimedia Appendix 4).

Differences in additional sleep metrics listed in Multimedia Appendix 1 are detailed in Multimedia Appendix 4. Although statistically significant differences (*P* >.05 in all cases) were observed in sleep stage metrics (light, deep, and REM sleep) between the 2 algorithms, the magnitude of these differences was modest (<5 minutes). Similar patterns in sleep metrics were found when analyses were repeated using a 3-level categorization of variation from typical sleep patterns (Multimedia Appendix 5), and when excluding participants with fewer than 4 hours of sleep data on more than 30% of days (results not shown).

## Discussion

### Principal Findings

We developed and implemented a user-centric algorithm (TSP) to more accurately classify primary sleep logs based on individuals’ habitual sleep patterns, using sequence-level Fitbit data from the All of Us Researcher Workbench. The TSP algorithm reclassified approximately 401,777 out of 8,435,172 (4.76%) sleep logs—originally labeled as nonprimary under the default calendar-relative isMainSleep algorithm—as primary sleep, thereby better capturing short sleep segments occurring within users’ primary sleep period, particularly among individuals with disrupted sleep patterns. Compared with TSP, the isMainSleep algorithm yielded lower estimates for key sleep metrics, including TATS, TST, WASO, total wakefulness duration, and NWAK, while producing higher estimates for nonprimary sleep counts and sleep efficiency percentage. As expected, differences in sleep stage and pattern metrics between the 2 algorithms were modest. These findings were consistent across empirically derived quartiles of variation from typical sleep patterns, as well as in a sensitivity analysis excluding participants with insufficient sleep data.

### Interpretation of Findings

Although the algorithm had no impact on 7,870,306 out of 8,435,172 (93.30%) sleep logs, we observed that participants with a higher proportion of “stitched” logs—indicative of disrupted or fragmented sleep—also had a higher incidence of insomnia. These findings suggest that, although the 2 algorithms may yield comparable results in the general healthy population, the isMainSleep algorithm may substantially misestimate sleep metrics among individuals with irregular sleep patterns or underlying sleep disorders such as insomnia. Notably, the American Academy of Sleep Medicine estimates that 30% of adults experience symptoms of insomnia [25]. Our prior work [3] highlighted the value of long-term monitoring of sleep stages, duration, and regularity in identifying chronic disease risk. These findings reinforce broader concerns about the limitations of traditional sleep monitoring methods (eg, 7-14-day actigraphy with sleep diaries and manual annotations) and emphasize the advantages of advanced, passive technologies for reliable, large-scale assessment of sleep-wake behavior [26,27]. Given the growing body of evidence suggesting that sleep regularity may be an even stronger predictor of health outcomes than sleep duration itself [28], obtaining an accurate, day-to-day picture of sleep-wake behaviors and disturbances—beyond the typical 1- to 2-week monitoring period—is essential for drawing valid inferences about the bidirectional relationship between sleep and health. Our method offers a particular advantage in this regard, as it is more sensitive to capturing irregular and fragmented sleep patterns that often lead to misclassification or failure in algorithms developed and validated primarily in generally healthy populations.

The user-centric algorithm represents a significant advancement in epidemiologic and sleep disorder–specific research using Fitbit-derived sleep data by aligning the identification of primary sleep periods with individuals’ homeostatic and physiological sleep patterns, rather than arbitrary calendar-based cut-offs. Using log “stitching” and “cleaning” procedures, the TSP algorithm has minimal impact on sleep staging metrics but substantially affects the computation of key sleep-wake metrics such as bedtime, wake time, TST, TATS, WASO, counts of nonprimary sleep, and sleep efficiency percentage. Notably, in the most affected population, the observed differences in these metrics approached approximately half of the minimum clinically important difference established in studies evaluating treatment responses for insomnia [29,30]. Consequently, this method enhances the precision of day-over-day behavioral sleep metrics and provides more relevant insights into the behavioral and physiological factors influencing sleep health—for most people some of the time, and for some people (likely those with chronic insomnia) much of the time. This refinement in preprocessing sleep data from wearables is critical for advancing our understanding of the complex interplay between longitudinal measurements of sleep-wake patterns and various health conditions [28,31].

Although statistically significant, differences in staged sleep durations between the 2 algorithms were modest and unlikely to be clinically meaningful. This is likely because most sleep logs reclassified from nonprimary to primary sleep and “stitched” by the TSP algorithm are less than 3 hours in duration and, therefore, do not include sleep staging information. As the TSP algorithm focuses on refining measures related to sleep-wake proportionality using existing sleep logs through data modeling, it does not address limitations inherent in the Fitbit sleep staging algorithm. For example, sleep logs that consist of 1 or more unstaged sleep segments are “stitched” with staged sleep segments by the TSP algorithm to form a complete primary sleep period. However, TSP does not update the reported sleep stages for the entire sleep period because the raw signal data used to estimate sleep stages are not available. This situation occurs relatively infrequently, affecting fewer than 15,029 (0.20%) TSP-classified primary sleep logs (N=7,518,329) in which both staged and unstaged sleep segments coexist. To further enhance the utility of the TSP algorithm, we provide a flag to identify all hybrid stitched TSP sleep logs (ie, those in which staged and unstaged sleep segments coexist), offering a simple way to filter out nights with potentially underestimated sleep architecture. This flag is important for sleep stage–based research because it helps identify sleep logs that are fully captured and staged by the default isMainSleep algorithm.

We found clinically meaningful differences for metrics related to sleep disturbances and duration—that is, the TSP algorithm achieved its intended goal of addressing issues related to sleep-wake proportionality, particularly among individuals with the most disrupted sleep patterns. Therefore, relying solely on the isMainSleep algorithm to identify primary sleep logs could lead to misaggregation of sleep data and inaccuracies in calculating sleep and wake durations, as well as day-over-day bed and wake time metrics. Both types of metrics have significant health implications [28,31]. As shown in our conceptual and data examples, the isMainSleep algorithm is more likely to misassign sleep to the incorrect day and misclassify primary sleep as nonprimary sleep (and vice versa), especially when sleep is fragmented on either side of midnight. Although the analysis and comparison conducted in this study were data-driven, future studies are needed to evaluate the performance of the TSP algorithm against polysomnography or actigraphy with sleep diaries, particularly among individuals with atypical sleep patterns or sleep disorders.

This study provides a practical guide for utilizing the user-centric sleep algorithm on a cloud-based platform and offers a user-friendly R package to streamline its implementation. Prior technical automation codes required self-reported sleep logs from participants [13], whereas the R package developed in this study relies solely on Fitbit sleep data from wearables. While the validity of passively detecting and reassigning logs to the primary sleep period was explored in this study, we hope the open-source R package will enable researchers to easily evaluate whether the reassigned logs adequately capture the sleep patterns of interest—thereby promoting transparency, reproducibility, and broader accessibility of Fitbit Web API data for clinical and translational research. These contributions align with open science principles [32,33], encouraging further refinement of sleep analysis methods and advancing sleep research. The algorithm’s applicability extends to all Fitbit devices, with the potential to generalize the logic of sleep-wake pattern identification to other data sources—such as manually annotated actigraphs and sleep data from other consumer wearable devices—by appropriately adapting the algorithm’s concept to accommodate the unique data types and representations of different sources. Although external validation is needed, this open-source R package equips researchers with a tool informed by both Fitbit developers and sleep medicine experts, fostering improved data interpretation and methodological advancement by addressing known limitations in current algorithms.

### Limitations

Although we have demonstrated the key advantages of the user-centric TSP algorithm in accounting for habitual sleep patterns, a few important caveats remain. First, the absence of sleep data from gold-standard measures such as polysomnography limits our ability to directly evaluate algorithm performance. Future research should address this by incorporating polysomnography or other validated sleep assessments to better assess the algorithm’s validity. Second, the user-centric TSP algorithm assumes that all periods of wakefulness imputed between “stitched” primary sleep logs are part of the same primary sleep period. However, we are unable to distinguish between instances when the user was intentionally awake and engaged in purposeful activity versus attempting to sleep during the imputed wake period. As a result, the TSP algorithm may introduce variations in measured activity levels due to differences in how sleep and wake periods are segmented. Research studies designed to examine the 24-hour Activity Cycle model [34], which considers the interrelatedness of various activity and sleep behaviors, may need to perform sensitivity analyses to account for these variations and validate findings under diverse conditions. Third, TSP does not address inherent limitations of wearable data collection that may introduce errors in sleep logs, including issues such as low battery power, device removal during sleep, or poor heart rate variability data quality that prevents accurate sleep staging. Given that the median duration of Fitbit sleep monitoring in this study was 4.2 years, outlier data from a small proportion of nights are unlikely to significantly influence the overall understanding of habitual sleep behaviors over time. Lastly, the generalizability of our results may be limited by the presence of atypical sleep schedules, such as polyphasic sleep or rotating shift work, which our methods are not designed to adequately characterize. However, a prior study leveraging All of Us Fitbit sleep data showed that fewer than 7% of weekdays had a sleep onset outside the traditional sleep window of 8:00 PM to 2:00 AM, suggesting a low prevalence of atypical sleep schedules in this cohort [3]. Future studies could explore alternative algorithms or incorporate additional data sources to better understand and address the effects of variations from typical sleep patterns. That said, it is important to highlight that this new algorithm’s approach—identifying the primary sleep period based on an individual’s typical sleep pattern, rather than relying on the longest sleep log ending on a given calendar day—is more likely to accurately capture and aggregate sleep logs that reflect a person’s true primary sleep period, even if it occurs at an atypical time of the day. This approach, however, assumes that the individual exhibits at least some regularity in their sleep schedule. Such regularity is necessary to (1) leverage longitudinal data to establish what is “usual” for that individual and (2) identify sleep logs that sufficiently overlap with the TSP-defined primary sleep window to be appropriately labeled. In the latter case, only the most extremely variable schedules—which would be physiologically difficult to sustain over extended periods and are generally considered unhealthy from a circadian rhythm standpoint—are likely to result in primary sleep sessions that have no overlap with an individual’s “usual” primary sleep period. In such cases, these sessions may not be assignable by the TSP algorithm. The All of Us Research Program has taken active steps to address diversity gaps in Fitbit data collection through the Wearables Enhancing All of Us Research study, which provides devices at no cost to participants from underrepresented communities. This initiative may offer future opportunities to reevaluate the algorithm’s performance in a more sociodemographically diverse population.

### Conclusions

In summary, the user-centric algorithm represents a significant advancement in the analysis of sleep-wake patterns by aligning more closely with individuals’ habitual sleep behaviors rather than relying on fixed calendar times. This algorithm captures the natural variability in sleep schedules, offering a more accurate tool for evaluating sleep metrics related to schedule, duration, and disturbances. The robustness of the user-centric algorithm enhances its utility in both clinical and translational research, where individualized and precise sleep assessments are essential. To support its widespread adoption, we have developed a comprehensive and user-friendly R package, enabling seamless integration of the TSP algorithm into existing research workflows. This tool holds considerable potential for advancing personalized sleep medicine and deepening our understanding of sleep-related health outcomes.

## Appendix

Multimedia Appendix 1 Definitions and computations of key sleep metrics.

Multimedia Appendix 2 Technical implementation of the TSP algorithm.

Multimedia Appendix 3 Distribution of change in the mean number of primary sleep logs per night as a measure of variation from typical sleep patterns. Negative values represent participants whose non-primary sleep were mislabeled as primary sleep under isMainSleep. Values between 0 and 0.05 represent participants whose sleep logs were consistently classified under both algorithms. Values >0.05 represent participants whose sleep tends to be interrupted, and were previously incompletely captured under isMainSleep.

Multimedia Appendix 4 Descriptive stats of sleep metrics by calendar-relative (isMainSleep) and user-centric (TSP) algorithms by quartiles for variation from typical sleep patterns.

Multimedia Appendix 5 Descriptive stats of sleep metrics by calendar-relative (isMainSleep) and user-centric (TSP) algorithms by 3-level categories of variation from typical sleep patterns.

## Abbreviations

API: application programming interface
EHR: electronic health record
NWAK: number of awakenings
Q: quartile
REM: rapid eye movement
TATS: time attempting to sleep
TSP: typical sleep period
TST: total sleep time
WASO: wake after sleep onset

## Data Availability

To ensure the privacy of participants, All of Us data used for this study are available to approved researchers following registration, completion of ethics training, and attestation of a data use agreement through the All of Us Research Workbench platform, which can be accessed online [35]. However, the real-world data from one of the co-authors (LS) used in the example shown in Multimedia Appendix 3 are made available on GitHub [24].
