# Supplementary material for: Capturing Real-World Habitual Sleep Patterns With a Novel User-Centric Algorithm to Preprocess Fitbit Data in the All of Us Research Program: Retrospective Observational Longitudinal Study
Source: J Med Internet Res. 2025 Jul 28;27:e71718. doi: 10.2196/71718 (PMC12340457; doi:10.2196/71718)
Supplement: Multimedia Appendix 1 [file jmir_v27i1e71718_app1.docx]

| Metric | Conceptual definition | Technical implementation | | | |
| --- | --- | --- | --- | --- | --- |
|  |  | Calendar-relative “isMainSleep” algorithm | | | User-centric “TSP” algorithm |
| **Sleep Schedule** | | | | | |
| Primary Sleep Period | Period of time during which a typical person obtains the majority of their sleep | Based on the sleep log with the longest duration on each midnight-to-midnight calendar day | | Based on the median bedtime and wake times across all relevant sleep logs centered around a user’s median mid-sleep point | |
| Bedtime  (BT) | Start of the primary sleep period as algorithmically determined by Fitbit; may not directly reflect the intent to sleep | Start time of the first sleep log in the primary sleep period. | | | |
| Sleep Onset | Time of transition from wakefulness to sleep | Start time of the first sleep segment in the primary sleep period, with the first occurrence of a level that is not ‘wake’, ‘awake’, or ‘restless’. | | | |
| Sleep Offset | Time of the final awakening | End time of the final sleep segment in the primary sleep period, after which there are no more levels that are not ‘wake’, ‘awake’, or ‘restless’. | | | |
| Wake time (WT) | End of the primary sleep period; corresponds to an “out of bed” or “lights on” time | End time of the final sleep log in the primary sleep period. | | | |
| Time attempting to sleep (TATS) | Duration of the primary sleep period between a user’s bedtime and wake time for a given day. This includes all sleep logs as well as any intervening non-sleeping time between sleep logs that are not captured by a sleep log | Duration between BT and WT | | | Duration between BT of the first sleep log and WT of the final sleep log, including any intervening non-sleep log time. |
| Total sleep period duration | Duration between a user’s sleep onset and sleep offset for a given day. | Sleep offset datetime - sleep onset datetime | | | |
| Midsleep point (MSP) | Midpoint time between sleep onset and sleep offset | (Sleep onset datetime + sleep offset datetime) / 2 | | | |
| **Sleep Duration (within Primary Sleep Period)** | | | | | |
| Total sleep time (TST) in primary sleep period | Total time spent asleep during the primary sleep period | Total duration of all sleep segments, where the level of the sleep segment is not ‘awake’, ‘wake’, or ‘restless’ | | | |
| Total wakefulness duration | Total time spent awake during the primary sleep period | Total duration of all sleep segments, where the level of the sleep segment is ‘awake’, ‘wake’, or ‘restless’ | | Total duration of all sleep segments, where the level of the sleep segment is ‘awake’, ‘wake’, or ‘restless’, in addition to any periods of ‘imputed wake’ between sleep logs. | |
| Sleep efficiency percentage | Total time spent asleep as a proportion of the primary sleep period | (TST /TATS) *100. | | | |
| Sleep onset latency (SOL) | Time between bedtime and sleep onset | Sleep Onset - BT  Note: Because sleep logs are algorithmically initiated, it is often 0 for automatically created sleep logs. | | | |
| Latency from wake to end of log | Time between the final awakening to wake time | WT - Sleep Offset | | | |
| **Sleep Disturbances (within Primary Sleep Period)** | | | | | |
| Wake after sleep onset (WASO) | Total time spent awake after sleep onset and before the final awakening. Note: only includes wake periods > 3 minutes. | Total duration of all ‘wake’, ‘awake’ and ‘restless’ sleep segments between sleep onset and sleep offset | | | Total duration of all ‘wake’, ‘awake’ and ‘restless’ sleep segments between sleep onset and sleep offset, as well as intervening non-sleep time between sleep logs |
| Number of awakenings (NWAK) | Total number of contiguous periods spent awake after sleep onset. Note: only includes wake periods > 3 minutes. | Count of all contiguous ‘wake’, ‘awake’ or ‘restless’ sleep segments between sleep onset and sleep offset | | | Count of all contiguous ‘wake’, ‘awake’ and ‘restless’ sleep segments between sleep onset and offset, as well as intervening non-sleep periods between sleep logs |
| Number of long awakenings | Total number of contiguous periods spent awake >=30 minutes after sleep onset, as a proxy measure of a meaningful awakening. | Count of all contiguous wake, awake and restless sleep segments >=30 minutes in duration between sleep onset and sleep offset | | | Count of all contiguous wake, awake and restless sleep segments between sleep onset and sleep offset, as well as any intervening non-sleep log periods between sleep logs >=30 minutes in duration |
| Length of longest wake | Duration of the longest contiguous period spent awake after sleep onset. Note: only includes wake periods > 3 minutes. | Maximum duration among all contiguous wake, awake and restless sleep segments between sleep onset and sleep offset | | | Maximum duration among contiguous wake, awake and restless sleep segments between sleep onset and sleep offset, as well as any intervening non-sleep log periods between sleep logs |
| Non-primary Sleep Period) | | | | | |
| Count of non-primary sleep period | Number of sleep logs outside of primary sleep period | Count of all sleep logs labeled “isMainSleep=false” that end on a calendar day | | | Count of all sleep logs labeled “TSP=false” that end prior to next BT |
| Total sleep time in non-primary sleep period (TST-NP) | Total duration of sleep across sleep logs outside of the primary sleep period | Total duration of all sleep logs labeled “isMainSleep=false” that end on a calendar day | | | Total duration of all sleep logs labeled “TSP=false” that end prior to next BT |
| **Sleep Pattern and Stages** | | | | | |
| Periods of wakefulness | | | | | |
| Awake | Total time spent awake (classic sleep) across all sleep segments. | Total duration of all sleep segments with level “Awake” | | | |
| Restless | Total time spent restless (classic sleep) across all sleep segments. | Total duration of all sleep segments with level “Restless” | | | |
| Wake | Total time spent in wake (staged sleep) across all sleep segments. | Total duration of all sleep segments with level “Wake” | | | |
| Imputed wake | Period of time spent awake between sleep logs | Not applicable | Total duration of intervening time between “TSP=true” sleep logs | | |
| Periods of sleep | | | | | |
| Asleep | Total time spent asleep (classic sleep) across all sleep segments. | Total duration of all sleep segments with level “Asleep” | | | |
| Light | Total time spent in light stage sleep across all sleep segments. | Total duration of all sleep segments with level “Light” | | | |
| Deep | Total time spent in deep stage sleep across all sleep segments. | Total duration of all sleep segments with level “Deep” | | | |
| REM | Total time spent in REM stage sleep across all sleep segments. | Total duration of all sleep segments with level “REM” | | | |
