# Supplementary material for: Capturing Real-World Habitual Sleep Patterns With a Novel User-Centric Algorithm to Preprocess Fitbit Data in the All of Us Research Program: Retrospective Observational Longitudinal Study
Source: J Med Internet Res. 2025 Jul 28;27:e71718. doi: 10.2196/71718 (PMC12340457; doi:10.2196/71718)
Supplement: Multimedia Appendix 2 [file jmir_v27i1e71718_app2.docx]

**Technical implementation of the TSP algorithm:**

The implementation steps outlined below are applicable to sleep data captured from all Fitbit devices.

*Step 1: Construct Fitbit sleep logs from sequence-level sleep data*

Currently, the Fitbit sequence level sleep data available on All of Us Researcher Workbench only includes the start date time and duration for each sleep segment. Therefore, to implement the user-centric TSP algorithm on All of Us Researcher Workbench, we first apply a stitching rule to the sequence level sleep data to reconstruct Fitbit sleep logs by aggregating consecutive sleep segments that are separated by less than one hour. While this simplified approach may not perfectly replicate the structure of algorithmically-constructed Fitbit sleep logs, the discrepancy is likely minimal as our post hoc QA (quality assurance) process identified data inconsistencies in only <0.2% of sleep logs (e.g.sleep logs reporting more than 1 value for “isMainSleep”). This step of re-constructing sleep logs can be omitted, however, if the sleep data is accessed directly from the Fitbit Web API and includes the logID data element. In this case, sleep logs are simply those sequence level data that share a common logID.

*Step 2: Identify the typical sleep period*

Next, we calculate the mid-sleep point (MSP) as the midpoint time between sleep onset and sleep offset for each isMainSleep-classified primary sleep log. We determine the median MSP across all available “isMainSleep=true” logs for each user, and also establish a time range of median mid-sleep point + /- 8 hours as the overlap period over which sleep logs are deemed relevant for establishing the user’s typical sleep period. Lastly, each user’s TSP bedtime and TSP wake time are calculated as the median bedtime and wake time across all relevant sleep logs, with bedtimes derived from the start of the first relevant sleep log in each sleep period and wake times derived from the end of the last relevant sleep log in each sleep period.

*Step 3: Classify sleep logs based on overlap with the typical sleep period*

All sleep logs that overlap with the typical sleep period, i.e. with bedtime (BT) and wake time (WT) between the TSP bedtime and wake time as defined above, are classified as primary sleep (“TSP=true”). All other sleep logs that do not meet the above criteria are classified as non-primary sleep (“TSP=false”), as these are most likely non-primary sleep outside of the typical sleep period.

Given how inconsistent sleep schedules may lead to incorrect BT /WT estimates, if the time between the TSP BT and WT is <5 hrs (i.e., indicative of habitually unhealthy short sleep durations), then the TSP BT and WT are set as the 7-hour window of sleep opportunity around the median MSP, based on the minimum recommended sleep opportunity for adults[1,2] (e.g., BT=MSP-3.5hrs and WT=MSP+3.5hrs). Though we acknowledge this as a simplifying assumption, the prevalence of this edge case was relatively low (<1% of sleep logs, n = 69,067), and contributed by <5% of the total analytical sample (n= 417). In addition to BT and WT, these TSP-classified primary sleep logs can now be used to calculate other metrics related to a user’s sleep schedule, sleep duration, and sleep disturbances.

References:

1. Consensus Conference Panel, Watson NF, Safwan Badr M, Belenky G, Bliwise DL, Buxton OM, et al. Recommended Amount of Sleep for a Healthy Adult: A Joint Consensus Statement of the American Academy of Sleep Medicine and Sleep Research Society. J Clin Sleep Med. 2015 [cited 25 Sep 2024]. doi:10.5664/jcsm.4758
2. Hirshkowitz M, Whiton K, Albert SM, Alessi C, Bruni O, DonCarlos L, et al. National Sleep Foundation’s sleep time duration recommendations: methodology and results summary. Sleep Health. 2015;1: 40–43. doi:10.1016/j.sleh.2014.12.010
