# Supplementary figures and images for: Capturing Real-World Habitual Sleep Patterns With a Novel User-Centric Algorithm to Preprocess Fitbit Data in the All of Us Research Program: Retrospective Observational Longitudinal Study
Source: J Med Internet Res. 2025 Jul 28;27:e71718. doi: 10.2196/71718 (PMC12340457; doi:10.2196/71718)

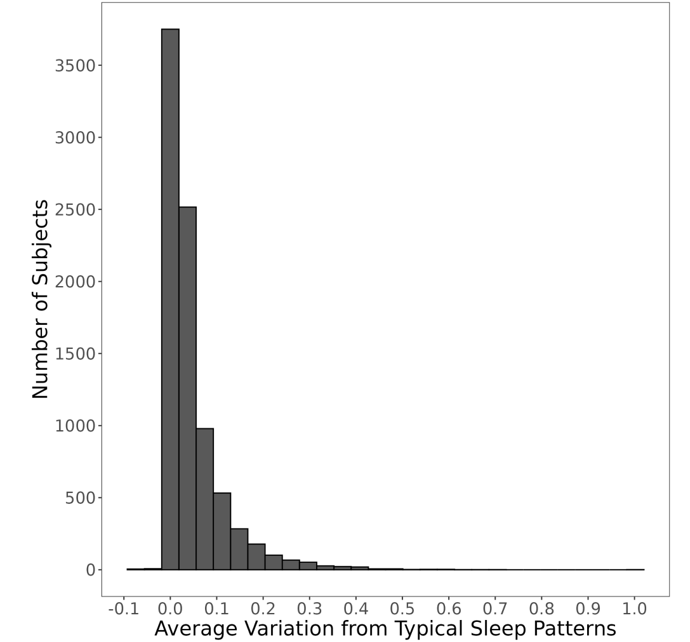

Supplement: Multimedia Appendix 3 [file jmir_v27i1e71718_app3.png]
