# Supplementary material for: Capturing Real-World Habitual Sleep Patterns With a Novel User-Centric Algorithm to Preprocess Fitbit Data in the All of Us Research Program: Retrospective Observational Longitudinal Study
Source: J Med Internet Res. 2025 Jul 28;27:e71718. doi: 10.2196/71718 (PMC12340457; doi:10.2196/71718)
Supplement: Multimedia Appendix 4 [file jmir_v27i1e71718_app4.docx]

|  | | | | | | | | |
| --- | --- | --- | --- | --- | --- | --- | --- | --- |
| **Variable** | **Algorithm** | **Q1** | **Q2** | **Q3** | **Q4** | **P-value (A^)** | **P-value (B^^)** | **P-value (AxB^^)** |
| Mean # logs / day (Range) | NA | [-0.0761,0.00735] | [0.00735,0.0235] | [0.0235,0.0594] | [0.0594,1] |  |  |  |
| Number of participants | NA | 2140 | 2141 | 2141 | 2141 |  |  |  |
| Number of logs | isMainSleep | 1428784 | 2122928 | 2042406 | 1711392 | NA | NA | NA |
|  | TSP | 1408141 | 2118034 | 2080945 | 1902153 |  |  |  |
| Person-days | isMainSleep | 1427450 | 2132910 | 2063404 | 1756258 | NA | NA | NA |
|  | TSP | 1420310 | 2123700 | 2051308 | 1738234 |  |  |  |
| Sleep schedule | | | | | | | | |
| Bedtime (BT) | isMainSleep | 22:50:12 [22:01:54,23:36:00] | 23:06:18 [22:27:12,23:51:54] | 23:40:36 [22:45:24,00:46:12] | 23:13:36 [22:27:12,00:04:06] | <0.0001 | 0.9465 | <0.0001 |
|  | TSP | 22:54:06 [22:08:24,23:39:54] | 23:09:00 [22:30:00,23:55:48] | 23:33:48 [22:38:06,00:42:30] | 23:15:12 [22:28:24,00:06:36] |  |  |  |
| Sleep onset | isMainSleep | 22:55:54 [22:07:24,23:41:12] | 23:10:54 [22:32:12,23:56:54] | 23:43:06 [22:49:36,00:49:00] | 23:18:24 [22:31:18,00:08:30] | <0.0001 | <0.0001 | <0.0001 |
|  | TSP | 23:00:06 [22:14:54,23:45:48] | 23:13:36 [22:35:24,00:00:42] | 23:37:42 [22:41:12,00:46:18] | 23:19:24 [22:32:54,00:11:06] |  |  |  |
| Sleep offset | isMainSleep | 06:16:30 [04:46:00,06:59:24] | 06:28:36 [05:47:00,07:06:48] | 05:56:18 [04:48:00,06:45:36] | 06:17:54 [05:23:12,06:59:30] | <0.0001 | <0.0001 | <0.0001 |
|  | TSP | 06:31:36 [05:41:48,07:12:36] | 06:44:30 [06:08:24,07:19:42] | 06:33:42 [05:42:36,07:20:24] | 06:39:18 [05:55:30,07:17:24] |  |  |  |
| Wake time (WT) | isMainSleep | 06:19:54 [04:49:24,07:03:12] | 06:32:06 [05:49:36,07:10:06] | 05:59:06 [04:50:24,06:48:30] | 06:20:36 [05:25:42,07:02:12] | <0.0001 | <0.0001 | 0.0001 |
|  | TSP | 06:34:54 [05:44:24,07:15:36] | 06:47:24 [06:10:54,07:22:48] | 06:36:30 [05:44:18,07:22:12] | 06:42:00 [05:58:12,07:20:48] |  |  |  |
| Time attempting to sleep (TATS) | isMainSleep | 455.29 (401.05, 490.54) | 456.37 (421.53, 485.75) | 441.44 (400.32, 476.44) | 404.72 (350.4, 447.91) | <0.0001 | 0.435 | <0.0001 |
|  | TSP | 466.23 (425.13, 499.34) | 465.32 (433.67, 494.25) | 457.55 (420.89, 490.86) | 442.03 (399.17, 482.93) |  |  |  |
| Total sleep duration | isMainSleep | 446.63 (391.27, 480.3) | 447.18 (413.27, 476.94) | 433.84 (393.46, 468.08) | 398.74 (345.23, 441.66) | <0.0001 | <0.0001 | <0.0001 |
|  | TSP | 457.05 (416.54, 489.53) | 456.64 (425, 485.36) | 449.63 (412.56, 482.44) | 435.74 (393.1, 476.47) |  |  |  |
| Midsleep point (MSP) | isMainSleep | 02:32:42 [01:31:12,03:14:24] | 02:48:36 [02:14:30,03:27:54] | 02:49:12 [01:58:12,03:34:42] | 02:47:12 [02:01:42,03:28:00] | <0.0001 | <0.0001 | <0.0001 |
|  | TSP | 02:46:48 [02:04:36,03:25:00] | 02:58:12 [02:25:42,03:36:36] | 03:04:06 [02:20:18,03:51:48] | 03:00:18 [02:18:30,03:40:24] |  |  |  |
| Sleep duration | | | | | | | | |
|  |  |  |  |  |  |  |  |  |
| TST of primary sleep period | isMainSleep | 419.97 (363.65, 452.04) | 418.46 (385.85, 446.26) | 401.71 (363.19, 434.31) | 365.73 (313.6, 406.82) | <0.0001 | <0.0001 | <0.0001 |
|  | TSP | 429.42 (389.58, 459.34) | 425.48 (394.92, 453) | 413.39 (376.88, 445.1) | 387.15 (342.55, 428.13) |  |  |  |
| Daily TST | isMainSleep | 408.84 (345.53, 442.93) | 411.5 (378.32, 440.16) | 394.45 (355.59, 426.41) | 357.78 (305.42, 398.66) | <0.0001 | <0.0001 | <0.0001 |
|  | TSP | 417.96 (375.21, 448.83) | 419.32 (387.54, 445.49) | 405.34 (369.6, 436.39) | 379.1 (333.34, 419.58) |  |  |  |
| Total wakefulness duration | isMainSleep | 33.1 (25.13, 41.39) | 35.34 (29.24, 42.38) | 36.19 (29.72, 43.65) | 34.79 (27.05, 42.68) | <0.0001 | <0.0001 | 0.1601 |
|  | TSP | 33.45 (25.5, 41.81) | 36.9 (30.58, 43.98) | 40.11 (33.67, 48.29) | 49.01 (40.03, 59.93) |  |  |  |
| Sleep efficiency percentage | isMainSleep | 92.54 (90.96, 93.85) | 92.21 (90.85, 93.32) | 91.72 (90.31, 92.9) | 91.27 (89.66, 92.67) | <0.0001 | <0.0001 | <0.0001 |
|  | TSP | 92.48 (90.9, 93.8) | 91.98 (90.59, 93.08) | 91.03 (89.65, 92.22) | 88.96 (86.71, 90.51) |  |  |  |
| Sleep onset latency (SOL) | isMainSleep | 4.73 (2.99, 6.73) | 4.44 (3.11, 6.18) | 3.88 (2.7, 5.52) | 3.11 (2.05, 4.52) | <0.0001 | <0.0001 | <0.0001 |
|  | TSP | 4.88 (3.09, 6.95) | 4.51 (3.17, 6.24) | 3.93 (2.73, 5.58) | 3.13 (2.09, 4.56) |  |  |  |
| Latency from wake to end of log | isMainSleep | 2.99 (1.46, 4.54) | 3.13 (2.04, 4.48) | 2.83 (1.83, 4.12) | 2.41 (1.48, 3.66) | <0.0001 | <0.0001 | 0.1601 |
|  | TSP | 3.03 (1.5, 4.59) | 3.1 (2.02, 4.45) | 2.73 (1.77, 3.99) | 2.28 (1.36, 3.51) |  |  |  |
| Sleep Disturbances | | | | | | | | |
|  |  |  |  |  |  |  |  |  |
| Wake after sleep onset (WASO) | isMainSleep | 28.73 (22.07, 36.3) | 31.25 (25.88, 37.53) | 32.99 (26.95, 40.02) | 33.06 (26.42, 40.91) | <0.0001 | <0.0001 | <0.0001 |
|  | TSP | 29.96 (23.23, 37.9) | 33.34 (27.94, 39.91) | 37.73 (31.91, 45.21) | 48.6 (40.28, 59.68) |  |  |  |
| # of awakenings | isMainSleep | 4.3 (3.25, 6) | 4.9 (3.82, 6.57) | 5.07 (3.98, 6.83) | 4.81 (3.72, 6.41) | <0.0001 | <0.0001 | <0.0001 |
|  | TSP | 4.42 (3.36, 6.24) | 5.02 (3.95, 6.71) | 5.3 (4.21, 7.11) | 5.36 (4.21, 7.08) |  |  |  |
| # of long awakenings | isMainSleep | 0.03 (0.01, 0.06) | 0.04 (0.02, 0.07) | 0.05 (0.03, 0.08) | 0.06 (0.03, 0.11) | <0.0001 | <0.0001 | <0.0001 |
|  | TSP | 0.03 (0.01, 0.06) | 0.05 (0.04, 0.08) | 0.09 (0.06, 0.12) | 0.18 (0.13, 0.26) |  |  |  |
| Length of longest wake | isMainSleep | 11.8 (9.28, 14.09) | 12.56 (10.66, 14.43) | 13.05 (10.98, 15.19) | 13.58 (11.07, 16.09) | <0.0001 | <0.0001 | <0.0001 |
|  | TSP | 12.45 (9.97, 14.71) | 14.13 (12.07, 16.03) | 16.91 (14.58, 19.35) | 25.35 (21.08, 31.95) |  |  |  |
| Sleep pattern and stages | | | | | | | | |
|  |  |  |  |  |  |  |  |  |
| Wake | isMainSleep | 36.71 (31.43, 43.15) | 38.06 (32.9, 44.07) | 39.32 (34.09, 45.33) | 38.78 (33, 45.48) | <0.0001 | <0.0001 | <0.0001 |
|  | TSP | 36.79 (31.48, 43.1) | 38.15 (33.06, 44.21) | 39.56 (34.32, 45.6) | 39.27 (33.4, 46.15) |  |  |  |
| Awake | isMainSleep | 5.02 (3.38, 8.04) | 5.8 (4.21, 7.95) | 6.19 (4.37, 8.6) | 6 (4.14, 8.68) | 0.0036 | 0.5199 | <0.0001 |
|  | TSP | 5.2 (3.5, 8.36) | 5.72 (4.15, 7.86) | 5.94 (4.2, 8.33) | 5.62 (3.75, 8.21) |  |  |  |
| Restless | isMainSleep | 16.66 (10, 26.41) | 20.21 (13.94, 28.63) | 21 (14, 30.14) | 19.2 (12.19, 29.45) | <0.0001 | 0.5711 | <0.0001 |
|  | TSP | 17.9 (10.75, 27.47) | 19.98 (13.82, 29.01) | 20.38 (13.39, 29.87) | 18.4 (11.19, 28.8) |  |  |  |
| Imputed Wake | isMainSleep | NA | NA | NA | NA | <0.0001 | NA | NA |
|  | TSP | 82.5 (65.5, 103.04) | 90.2 (74.53, 106.29) | 95.74 (81.8, 109.66) | 103.82 (90.72, 119.98) |  |  |  |
| Asleep | isMainSleep | 350.6 (205.15, 428.46) | 374.9 (280.11, 424.63) | 346.77 (246.34, 407.17) | 283.63 (184.52, 368.02) | <0.0001 | 0.3895 | <0.0001 |
|  | TSP | 377.01 (235.66, 439.15) | 373.5 (269.37, 426.06) | 340.99 (223.14, 406.85) | 265.68 (162.42, 359.97) |  |  |  |
| Light | isMainSleep | 273.6 (249.73, 300.41) | 273.28 (250.16, 298.37) | 269.38 (244.31, 294.84) | 256.5 (228.3, 284.74) | <0.0001 | <0.0001 | <0.0001 |
|  | TSP | 274.4 (250.42, 300.88) | 274.1 (251.22, 298.98) | 271.16 (245.81, 296.45) | 260.03 (232.42, 289.15) |  |  |  |
| Light as % of TST | isMainSleep | 63.77 (60.07, 68.46) | 64.15 (60.6, 68.42) | 65.3 (61.46, 69.52) | 67.63 (63.03, 71.95) | <0.0001 | <0.0001 | <0.0001 |
|  | TSP | 63.58 (59.96, 68.16) | 63.94 (60.35, 68.2) | 64.74 (60.9, 68.92) | 65.48 (61.4, 69.89) |  |  |  |
| Deep | isMainSleep | 65.73 (54, 75.9) | 64.22 (53.87, 74.37) | 60.54 (50.78, 70.05) | 53.12 (43.19, 63.54) | <0.0001 | <0.0001 | <0.0001 |
|  | TSP | 66.23 (54.68, 76.19) | 64.38 (54.01, 74.7) | 60.81 (51.11, 70.49) | 53.8 (43.79, 64.46) |  |  |  |
| Deep as % of TST | isMainSleep | 15.42 (12.99, 17.51) | 15.11 (12.84, 17.37) | 14.75 (12.47, 16.92) | 13.84 (11.7, 16.28) | <0.0001 | <0.0001 | <0.0001 |
|  | TSP | 15.45 (13.01, 17.52) | 15.06 (12.84, 17.35) | 14.66 (12.36, 16.8) | 13.46 (11.3, 15.9) |  |  |  |
| REM | isMainSleep | 89.6 (74.7, 102.82) | 88.11 (74.94, 101.05) | 83.64 (70.24, 96.81) | 73.85 (59.78, 87.84) | <0.0001 | <0.0001 | <0.0001 |
|  | TSP | 89.97 (75.39, 103.19) | 88.43 (75.35, 101.31) | 84.17 (70.69, 97.16) | 74.74 (60.71, 88.83) |  |  |  |
| REM as % of TST | isMainSleep | 20.82 (17.85, 23.19) | 20.7 (17.88, 23.04) | 20.13 (17.37, 22.41) | 18.98 (16.26, 21.32) | <0.0001 | <0.0001 | <0.0001 |
|  | TSP | 20.87 (17.93, 23.23) | 20.64 (17.86, 23.03) | 20.02 (17.27, 22.25) | 18.43 (15.7, 20.92) |  |  |  |
| Non-primary sleep period | | | | | | | | |
|  |  |  |  |  |  |  |  |  |
| Count per day | isMainSleep | 0.04 (0.02, 0.09) | 0.08 (0.05, 0.14) | 0.15 (0.1, 0.22) | 0.31 (0.22, 0.46) | <0.0001 | <0.0001 | <0.0001 |
|  | TSP | 0.06 (0.02, 0.15) | 0.07 (0.03, 0.15) | 0.1 (0.05, 0.2) | 0.15 (0.07, 0.27) |  |  |  |
| TST-NP | isMainSleep | 90.34 (78.04, 105.45) | 95.95 (84.27, 108.73) | 100.51 (87.87, 112.86) | 105.54 (91.02, 121.49) | <0.0001 | <0.0001 | <0.0001 |
|  | TSP | 97.5 (82, 120.85) | 100.02 (86.29, 119.12) | 100.96 (86.93, 120.86) | 101.93 (86.16, 123.33) |  |  |  |
| Note that the total number of logs across quartiles may not equal the total number of logs given the simplified approach of definition that was used to identify sleep logs and in these analyses we only consider nights where a non-waking level is present. A^ - Refers to main effect of quartile B^^ - Refers to main effect of algorithm AxB^^^ - Refers to interaction effect of quartile and algorithm | | | | | | | | |
